# Tyrosine kinase signaling pathways as therapeutic targets in autoimmune subepidermal blistering skin diseases (pemphigoid diseases)

**DOI:** 10.3389/fimmu.2026.1767876

**Published:** 2026-03-13

**Authors:** Simon Vikár, Attila Mócsai

**Affiliations:** 1 Department of Physiology, Semmelweis University School of Medicine, Budapest, Hungary; 2 HUN-REN–SU Inflammation Physiology Research Group, Hungarian Research Network and Semmelweis University, Budapest, Hungary; 3 MTA–SE “Lendület” Inflammation Physiology Research Group, Hungarian Academy of Sciences and Semmelweis University, Budapest, Hungary

**Keywords:** autoimmunity, blistering skin diseases, JAK, neutrophils, precision medicine, signalling, targeted therapy, tyrosine kinases

## Abstract

Pemphigoid diseases, such as bullous pemphigoid and epidermolysis bullosa acquisita, are severe organ-specific autoimmune diseases characterized by subepidermal skin blistering with increasing incidence in recent years. Although there have been substantial advances in understanding the pathomechanism of these diseases in the last decades, and the first specific therapy targeting the IL-4 and IL-13 pathway (dupilumab) has been approved by the FDA for bullous pemphigoid, further research is needed to eventually improve patient care. The characteristics of pemphigoid diseases include the formation of immune complexes and their recognition by Fcγ-receptors, as well as the development of a characteristic inflammatory cytokine microenvironment in the skin of the affected patients. Several non-receptor tyrosine kinases are involved in these events, playing a very important role in various signaling processes of immune cells. While certain Src-family kinases and the Syk tyrosine kinase play a very important role in signaling by Fcγ-receptors, JAK-family kinases are crucial players in the signaling of various cytokine receptors including, among others, the receptors of IL-4 and IL-13. The inhibition of these tyrosine kinases with small molecule inhibitors is an emerging therapeutic option in the treatment of an increasing number of immune-mediated diseases. Moreover, numerous studies have been conducted to examine proteins (including PLCγ2 and CARD9) in signal transduction following Fcγ-receptor activation in *in vitro* and *in vivo* experimental pemphigoid models, and an increasing number of case studies involving JAK inhibitors report the successful application of these drugs in various pemphigoid diseases. This review summarizes our current understanding of the therapeutically most promising tyrosine kinase signaling pathways in the pathogenesis of pemphigoid diseases.

## 1 Introduction

Pemphigoid diseases, such as bullous pemphigoid and epidermolysis bullosa acquisita, are rare, potentially life-threatening autoimmune blistering skin diseases with an increasing number of cases in recent years. A common feature of these diseases is the destruction of the dermo-epidermal junction, which results in painful, subepidermal blister formation on the skin and, in some cases (depending on the disease) in mucous membranes, leading to a severe decrease in quality of life (1). Despite intensive research in the last decades, the treatment of these diseases remains unresolved. Although clear progress has been made, as exemplified by the regulatory approval of dupilumab (by the FDA), the first targeted therapy for bullous pemphigoid, in 2025, general immunosuppressive therapy (mostly corticosteroids), with their often serious side effects (such as infections, hyperglycemia and cardiovascular complications), continues to be used as the first choice in the vast majority of cases (2, 3).

Non-receptor tyrosine kinases are intracellular enzymes that play a critical role in signal transduction processes associated with numerous cell surface receptors, including immunoreceptors (B-cell receptors, T-cell receptors and Fc-receptors), integrins (including complement receptors such as Mac-1/CR1) and cytokine receptors, through the phosphorylation of tyrosine residues of other proteins (4). Targeting these enzymes with small molecule inhibitors is an actively investigated therapeutic option, considering the reasonable advances of these inhibitors over biological therapies (oral-administration, shorter half-life, cost effectiveness). Initially, the research exploring the application of kinase inhibitors focused on certain hematological malignancies, while in the field of autoimmune diseases, they were first investigated in the treatment of inflammatory joint diseases (5). The latter line of research ultimately led to the regulatory approval of JAK inhibitors in rheumatoid arthritis, which represented a breakthrough in the treatment of immune-mediated diseases (6). In the following years, the investigation of various tyrosine kinase signaling pathways received substantial interest in other autoimmune diseases and became potentially attractive targets in autoimmune blistering skin diseases, as well (7). Studies from our research group and others suggested that tyrosine kinases associated with immunoreceptor activation, including certain Src-family kinases (8) and the Syk tyrosine kinase (9–12), play an important role in the development of pemphigoid diseases. Several lines of evidence also suggest a similar importance for JAK family tyrosine kinases, which have a crucial role in signaling by several cytokine receptors (13).

In this review, we summarize our current understanding of the role of immune receptor signaling pathways involved in the recognition of immune complexes formed during pemphigoid disease pathogenesis, as well as the role of JAK tyrosine kinases also intensively studied in pemphigoid diseases in the past years. First, we provide a brief overview of the characteristics of the most important pemphigoid diseases in experimental dermatology, highlighting the experimental data of the role of immune complexes and innate immunity. We then discuss the individual Fcγ-receptors and the role of tyrosine kinases in the related downstream signaling in the pathogenesis. Finally, we summarize our current knowledge regarding the use of JAK inhibitors in pemphigoid disease.

## 2 Pemphigoid diseases

### 2.1 General characteristics

**Figure 1:**
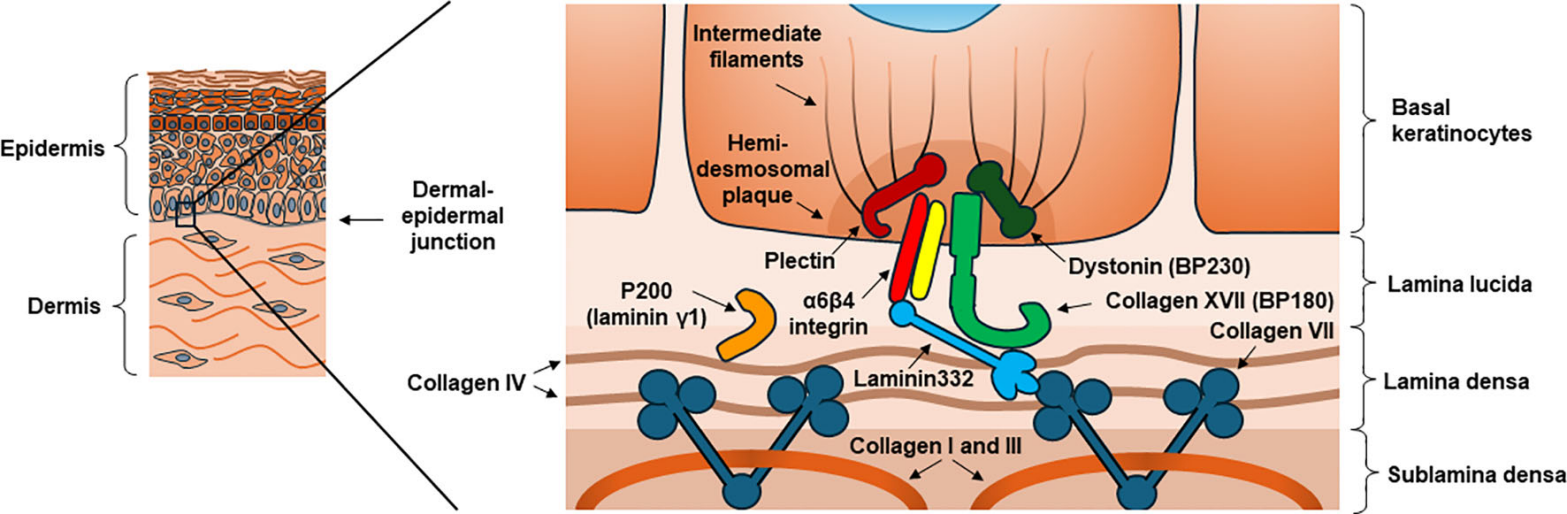
The dermo-epidermal junction in the skin, and the most common autoantigens in pemphigoid diseases. In some of these diseases hemidesmosomal proteins are the main targets of autoantibodies, such as collagen XVII (BP180) and often dystonin (BP230) in bullous pemphigoid. In other pemphigoid diseases, proteins of the basement membrane become targets of the pathogenic immune response, such as collagen VII in epidermolysis bullosa acquisita.

Autoimmune blistering skin diseases can be considered as model diseases of organ-specific autoimmunity. The individual diseases can be divided into two groups, pemphigoid and pemphigus diseases, which share the common feature that autoantibodies are produced against important anchoring proteins in the skin during the autoimmune process, eventually leading to blister formation (1). In case of the pemphigoid group, the antigens targeted by these antibodies are either hemidesmosomal proteins responsible for the attachment of the basal keratinocyte layer to the basement membrane, or certain anchoring proteins located in the basement membrane (**Figure 1**). These autoantigens differ in the distinct types of pemphigoid diseases (**Table 1**). The most common candidate of this disease group is bullous pemphigoid (BP), for which epidemiological studies report 4 to 22 new cases per one million population per year in Europe (14–16). In BP the most common targets of the autoantibodies are two hemidesmosomal proteins, the transmembrane collagen XVII (formerly known as BP180), and often the intracellular dystonin (formerly known as BP230). In another disease of the pemphigoid group, epidermolysis bullosa acquisita (EBA) which occurs much less frequently than BP but has been intensively studied in experimental dermatology in the previous decades, the autoantigen is the collagen VII protein, which is an important anchoring protein located in the basement membrane (1). The most important characteristics of the most common pemphigoid diseases are shown in **Table 1**, while **Figure 1** demonstrates the location of the most important autoantigens.

**Table 1:** Pemphigoid diseases and their models.

Disease	Autoantigen	Animal models	Human models
Bullous pemphigoid	Collagen XVII (BP180), dystonin (BP230)	Several passive (24, 57, 61) and active immunization (128, 129) models (antibodies against BP180 protein); lymphocyte transfer model (Autoreactive lymphocytes against BP180); *in vitro* neutrophil activation assay	Cryosection assay (28, 130), keratinocyte culture models (29), 3D skin equivalents (63) (Antibodies from BP patients); *in vitro* neutrophil activation assay
Epidermolysis bullosa acquisita	Collagen VII	Passive (131) and active immunization (59) models (antibodies against Collagen VII protein); *in vitro* neutrophil activation assay	Cryosection assay (132, 133) (Antibodies from EBA patients); *in vitro* neutrophil activation assay
Mucus membrane pemphigoid	Collagen XVII (BP180), dystonin (BP230) LAD-1, Laminin332, α6β4 integrin	Passive immunization model (55) (Antibodies against laminin332); *in vitro* neutrophil activation assay	*in vitro* neutrophil activation assay
Linear IgA-dermatosis	LAD-1, LABD97	Passive immunization model (134) (Antibodies against LABD97); *in vitro* neutrophil activation assay	*in vitro* neutrophil activation assay
Pemphigoid gestationis	Collagen XVII (BP180), dystonin (BP230)	*in vitro* neutrophil activation assay	*in vitro* neutrophil activation assay
Anti-p200 pemphigoid	p200 (lamini-γ1)	*in vitro* neutrophil activation assay	*in vitro* neutrophil activation assay
Lichen planus pemphigoid	Collagen XVII (BP180), dystonin (BP230)	*in vitro* neutrophil activation assay	*in vitro* neutrophil activation assay

In the first phase of these diseases, the pathogenic autoantibodies are formed and deposited in the appropriate layer of the skin as consequence of a loss of immune tolerance against the skin proteins. In this process, genetic studies imply that certain HLA haplotypes can play a role causing genetic susceptibility (17). Moreover, some drugs like DPP4 inhibitors (18) as well as immune checkpoint inhibitors (19) can take part of the induction of autoantibody formation resulting in a BP-like phenotype, however the exact mechanism of the blister-formation induced by these drugs is not fully elucidated. While the inhibition of DPP4 in the skin might result in elevated cytokine levels (CCL11/eotaxin) primarily inducing eosinophil recruitment (20), immune-checkpoint inhibitors can contribute to the incomplete elimination of autoreactive T cells inducing autoimmunity most frequently affecting the skin (21). Additionally to these factors, changes in the microbiome of the intestinal tract and skin (and the interplay between the microbiome of these two areas) may also play a role in BP and is an intensively researched area in recent years (22, 23). In contrast to the first phase, the processes following the deposition of the antibodies, leading to the formation of characteristic tight blisters, are generally referred to as the effector phase of the diseases. Antibody deposition and the consequent complement activation, which can be detected by direct immunofluorescence (and remain one of the most important diagnostic pillars in pemphigoid diseases) is normally followed by the infiltration of leukocytes, as well as dermo-epidermal separation, eventually leading to the formation of tense blisters (1). The crucial role played by antibodies in the pathomechanism is well demonstrated by the fact that injecting mice with antibodies against human collagen XVII or collagen VII, or antibodies produced in mammals against analogous mouse proteins result in severe blistering in the treated animals (24–27). In addition, it is also known that antibodies isolated from the blood of BP or EBA patients can cause separation of the dermo-epidermal junction in the cryosections of normal human skin in the presence of granulocytes and human blood plasma (12, 28). Several types of immune cells are supposed to play an important role in this dermo-epidermal separation and the resulting blister formation. In this review, we will focus in more detail on the role of the innate immune system.

### 2.2 Innate immune cells in the effector phase and in dermo-epidermal separation

**Figure 2:**
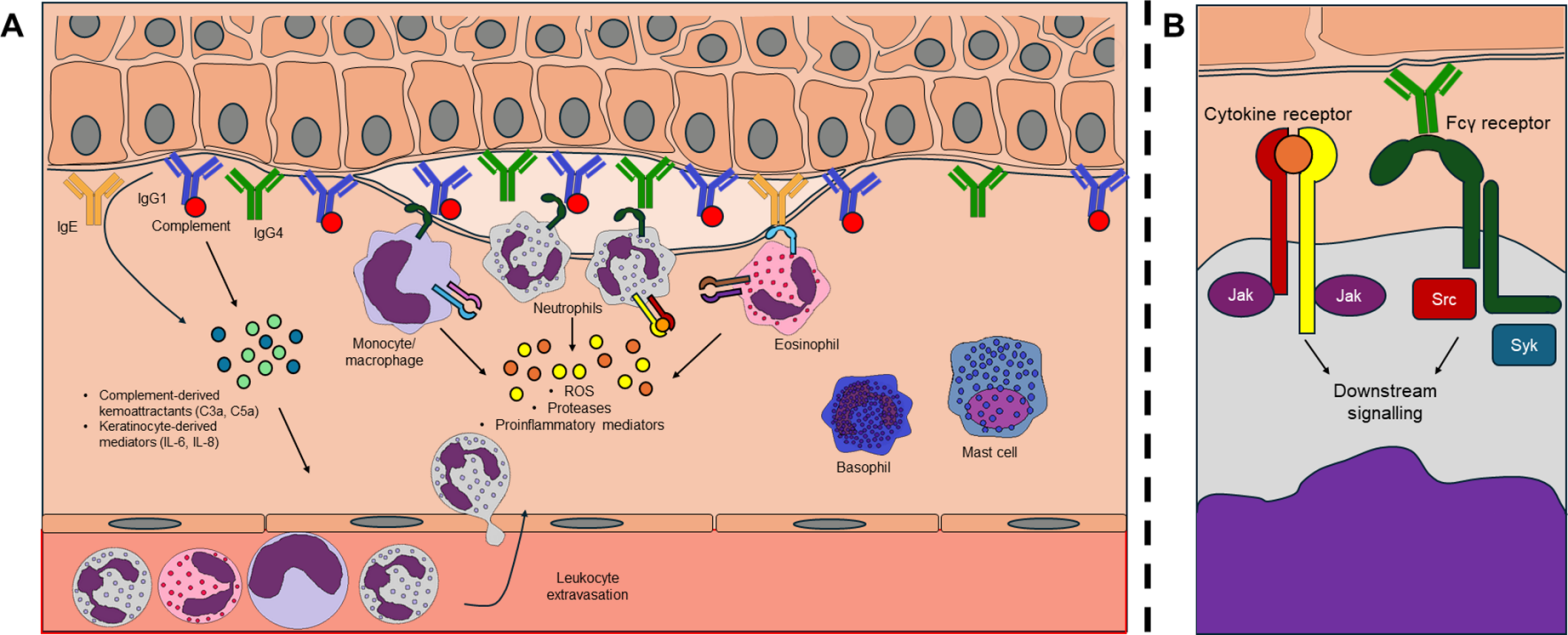
Blister formation in pemphigoid diseases. **(A)** during the development of bullous pemphigoid and epidermolysis bullosa acquisita, pathogenic autoantibodies are formed and deposited at the dermo-epidermal junction of the skin. The deposited antibodies can have a direct effect on basal keratinocytes and activate the complement system, leading to the release of pro-inflammatory mediators, which induces the extravasation of circulating myeloid cells. These cells recognize the immune complexes through their Fc receptors resulting in various effector responses (ROS, proteases, cytokines) ultimately leading to the separation of the dermo-epidermal junction. **(B)** in the processes of immune complex-recognition (via Fcγ receptors) and the detection of cytokines (via cytokine receptors), several non-receptor tyrosine kinase enzymes (JAK family, SRC family, Syk) are involved.

In pemphigoid diseases, the autoantibody deposition and the resulting immune complex formation is followed by the activation of the complement system, and there is increasing evidence supporting the importance of the direct activating effect of antibodies on keratinocytes, resulting in the release of inflammatory cytokines and chemokines (such as IL-6 and IL-8) (29). The release of inflammatory and chemotactic molecules leads to the infiltration of leukocytes. The appearance of this inflammatory cell infiltrate is followed by the separation of the dermo-epidermal junction, which is a result of the coordinated interaction of many different cell types (**Figure 2**). The role of several different leukocyte subsets in this process has been studied in various mouse and human experimental models, as well as in diseased patients.

In several BP and EBA experimental models, neutrophilic granulocytes (neutrophils) appear to be one of the most important cell types underlying dermo-epidermal separation. The active role of these cells in triggering dermo-epidermal separation is supported by the fact that, in the most widely used experimental model of EBA, mice treated with anti-collagen VII antibodies are completely protected in the case of neutrophil depletion or a neutrophil-deficient phenotype (30, 31), while neutrophil-depleted animals are also protected against the development of experimental BP induced by anti-BP180 IgG (32). In addition, in EBA experiments, mice are also protected against severe blistering in the absence of NADPH oxidase, which has a crucial role in the production of reactive oxygen species (ROS), an effector mechanism associated with neutrophil activation (30). Human ex vivo experiments have also shown that neutrophil granulocytes can induce dermo-epidermal separation. In this experimental model, although the degree of dermo-epidermal separation was increased when other leukocytes were also present, neutrophils alone caused the greatest degree of skin separation, and in this model the tissue damage could also be decreased by inhibiting ROS production (30). Furthermore, there was no BP IgG or EBA IgG induced skin separation observed in case of the inhibition of neutrophil proteases (neutrophil elastase, MMP9) (33). These proteases can have a pivotal role in physical blister formation as neutrophil elastase and MMP9 can cleave several components of the basement membrane as well as BP180 itself (34, 35).

In addition to neutrophils, various experiments also support the important role of eosinophils and monocytes/macrophages in autoimmune skin blistering. In BP, eosinophils are often described as the most characteristic and prominent cell type in the inflammatory infiltrate (36) and have been demonstrated to be essential in the development of skin symptoms triggered by anti-BP180 IgE antibodies in a BP mouse model (37).These cells highly contribute to the inflammatory cytokine environment in the skin, inducing the recruitment and activation of neutrophils by the production of several proinflammatory and chemotactic mediators (IL‐1β, IL‐5, IL‐6, IL-8, TNFα) (38). In addition, it has been shown recently that under certain conditions (following a treatment with IL-5), eosinophils are also capable of actively inducing dermo-epidermal separation ex vivo in human skin samples treated with IgG-s from BP patients even in the absence of neutrophils (39). Monocytes also constitute a significant part of inflammatory cell infiltration in human BP, and in the ex vivo human model, monocytes also contributed to the development of dermo-epidermal separation by potentiating the effect of neutrophils (40). Furthermore, the role of different monocyte populations also seems to be important in experimental EBA (41).

There is much less information about the contribution of basophils and mast cells in these processes. Basophilic granulocytes are primarily thought to play a role in the production of IL-31 and itching associated with bullous pemphigoid (42). The contribution of mast cells to the development of skin symptoms is somewhat controversial: while mast cell degranulation to IgE immune complex stimuli is considered an important event promoting neutrophil chemotaxis, their role has been found to vary in importance in different BP models (43), and they appear to have no significant role in the EBA mouse model (44).

The process of dermo-epidermal separation and blister development therefore involves the activation and interplay of numerous immune cell types (**Figure 2A**). In these processes the activation of Fcγ-receptors, which are important in the recognition of IgG immune complexes, as well as the activation of different cytokine receptors, many of which signal through JAK kinases play an essential role at the molecular level (**Figure 2B**). Recently, an increasing number of experiments have demonstrated the important role of the immune receptor and JAK signaling pathways in EBA and BP disease models. The following section discusses these experimental results and clinical data supporting the significance of these two pathways.

## 3 The inhibition of Fcγ-receptor signaling in pemphigoid diseases

### 3.1 Immune complex recognition by Fcγ-receptors

The most characteristic antibodies in human BP and EBA belong to the IgG subtypes (especially IgG1 and IgG4) (45, 46). A number of Fcγ-receptor subtypes play a key role in the recognition of these antibodies and the concomitant leukocyte activation. Several of these receptors have been studied in various mouse and human disease models. Before going into those details, we briefly describe the various mouse and human Fcγ-receptors and the differences between the two species (47) (**Table 2**).

**Table 2:** Fcγ-receptors in pemphigoid diseases.

Species	Receptor	Affinity	Expression	Mechanism	Role in pemphigoid diseases
Mouse	FcγRI	High affinity to IgG2A and 2B, low affinity to IgG3	Monocyte-derived dendritic cells	ITAM phosphorylation, activation	No role has been shown (in MMP animal model, the loss of common γ-chain results in protection (55))
	FcγRIIB	Low affinity to IgG 1, 2A and 2B	B cells and all myeloid cell types	ITIM phosphorylation, inhibition	Protective role in EBA active mouse model (59)
	FcγRIII	Low affinity to IgG 1, 2A and 2B	NK cells and all myeloid cell types	ITAM phosphorylation, activation	Important role in BP mouse models (57) (in MMP animal model, the loss of common γ-chain results in protection (55)), important role in *in vitro* neutrophil activation assays (135)
	FcγRIV	High affinity to IgG2A and 2B	Monocytes and neutrophils	ITAM phosphorylation, activation	Important role in BP (57) and EBA (56) mouse models, (in MMP animal model, the loss of common γ-chain results in protection (55)), important role in *in vitro* neutrophil activation assays (56, 135)
	FcRn	High affinity to all IgG (at pH < 6.5)	B cells, monocytes, neutrophils, dendritic cells, endothelial cells, intestinal epithelium	Inhibition of IgG degradation	Important role in BP (61) and EBA mouse models (52)
Human	FcγRI	High affinity to IgG1, 3 and 4	Monocytes and dendritic cells, (inducible by neutrophils and mast cells)	ITAM phosphorylation, activation	No role in BP cryosection assay (58)
	FcγRIIA	Low affinity to all IgG	All myeloid cell types	ITAM phosphorylation, activation	Important role in cryosection assay and *in vitro* neutrophil activation assay (58)
	FcγRIIB	Low affinity to all IgG	B cells, dendritic cells, basophils (small percentage of neutrophils and monocytes)	ITIM phosphorylation, inhibition	Seems to have protective role in BP (60)
	FcγRIIC	Low affinity to all IgG	NK cells, monocytes, neutrophils	ITAM phosphorylation, activation	Can have an important role in BP cryosection assay (58)
	FcγRIIIA	High affinity to IgG 1, 3 and 4; Low affinity to IgG2	NK cells, monocytes	ITAM phosphorylation, activation	Seems to have an important role in BP cryosection assay and *in vitro* neutrophil activation assay (58)
	FcγRIIIB	Low affinity to IgG1, 3 and 4	Neutrophils	Effect on other receptors	Seems to have an important role in BP cryosection assay (58)
	FcRn	High affinity to all IgG (at pH < 6.5)	Monocytes, neutrophils, dendritic cells, endothelial cells, syncytiothrophoblasts	Inhibition of IgG degradation	Important role in BP model with 3D skin equivalents (61), promising effect in clinical studies with efgartigimod

#### 3.1.1 Mouse Fcγ receptors

There are a total of five receptors among the mouse Fcγ receptors, three of which are activating receptors: FcγRI, FcγRIII, and FcγRIV. The ligand-binding transmembrane α-chains of these receptors are noncovalently bound to a transmembrane adapter protein called Fc-receptor common γ-chain (FcR γ-chain or simply FcRγ), which contains an intracellular immunoreceptor tyrosine-based activation motif (ITAM) that can be phosphorylated by tyrosine kinases resulting in downstream signaling. These receptors differ in their IgG specificity: although all three receptors can bind IgG2a and IgG2b, only FcγRI can bind IgG3, while FcγRIII can bind IgG1. They also differ in their tissue expression: while FcγRI is specific to certain dendritic cells (monocyte-derived dendritic cells) and FcγRIV is limited to monocytes, macrophages, and neutrophils, FcγRIII shows broader expression and can be found in all myeloid cells, as well as on NK-cells. In addition to these activating receptors, mice also have an inhibitory FcγR, FcγRIIB, which contains an immunoreceptor tyrosine-based inhibition motif (ITIM) in its intracellular domain, which can be phosphorylated and has an inhibitory effect on the cell activation. This receptor shows specificity for IgG2A, IgG2B, and IgG3 types and shows broad expression, being found on the surface of all myeloid cells and also on certain lymphocytes. The fifth member of this receptor family is the so-called neonatal Fc receptor (FcRn), which can bind to all IgG types and plays a crucial role in the recirculation of IgG antibodies, preventing their intracellular degradation (48).

#### 3.1.2 Human Fcγ receptors

There are a total of seven different human Fcγ receptors, which, like the mouse receptors, also differ in their signal transduction, specificity, and expression. Of these, a total of four receptors, FcγRI, FcγRIIA, FcγRIIC, and FcγRIIIA are activating receptors. While FcγRIIA and FcγRIIC α-chains have an intracellular domain containing their own ITAM motifs, FcγRI and FcγRIIIA are, similarly to mouse activating receptors, associated with the ITAM-containing FcRγ chain. While all these receptors can bind IgG1 and IgG4 subtypes, FcγRI is unable to recognize IgG2. It is known about their expression, that FcγRI is restricted to monocytes, macrophages, and dendritic cells, while it shows inducible expression in neutrophils and mast cells. FcγRIIA is expressed in all myeloid cells, FcγRIIC is present on NK cells, monocytes, and macrophages, and FcγRIIIA is constitutively expressed on neutrophils, eosinophils, basophils, and mast cells. In humans there is also an inhibitory receptor, FcγRIIB, which can recognize all IgG subtypes and is expressed primarily on B cells, basophilic granulocytes, and a small percentage of monocytes and neutrophils. In addition, there is a receptor without an intracellular domain (bound to the membrane by a GPI anchor), FcγRIIIB, which recognizes IgG1, 2, and 4 subtypes and is expressed only on neutrophils. Although it is not capable of independent signal transduction due to the absence of an ITAM/ITIM motif, its ligand binding may indirectly contribute to the activation processes of neutrophils (ROS production, phagocytosis). Finally, analogous to mouse receptors, the neonatal Fc-receptor (FcRn) also exists in humans, which is involved in the recirculation of IgG antibodies and the prevention of their intracellular degradation.

#### 3.1.3 Fcγ receptors in pemphigoid diseases

Pemphigoid diseases (unlike pemphigus) are known as Fc-dependent diseases. The importance of antibody recognition by Fcγ-receptors is well demonstrated by the fact that antibodies containing only the F_(ab)2_ region normally fail to induce disease in passive animal models of pemphigoid diseases (49, 50). However, in some BP animal models, Fc-independent blistering has also been reported (51), suggesting that antibodies can exert certain effects without Fc-receptors e. g. through their direct action on keratinocytes (52). Interestingly, genetic studies investigating individual FcγR polymorphisms show that the low affinity variant of FcγRIIIA associates with bullous pemphigoid, which might be explained with the impaired macrophage-mediated clearance of the pathogenic autoantibodies (53, 54).

The significance of individual Fcγ-receptors has been investigated in EBA and several different BP models, as well as in an animal model of mucous membrane pemphigoid, a rare but particularly hard-to-treat variant of pemphigoid diseases (25, 27, 50, 55). In general, it can be said that among the activating receptors, the role of FcγRIV appears to be indispensable in the experimental passive model of EBA (56), while in various models of BP, FcγRIII and FcγRIV seems to play an important role in the pathogenesis (50, 57). The blistering in anti-Laminin 332 mucous membrane pemphigoid is completely abrogated in FcR γ-chain (FcRγ) deficiency, although the role of individual Fcγ-receptors has not yet been investigated (55). Based on experiments performed on frozen sections of human skin, the effects of FcγRIIA and FcγRIIIB appear to be essential in the process of skin separation triggered by granulocytes (58). FcγRI does not appear to be a significant pathogenic factor in either mouse or human receptors, while FcγRIIB had a protective effect against the development of the severe symptoms in an active EBA mouse model (59), which is also implied in a human case study, where a mutation in this receptor resulted in an aggressive BP phenotype (60). It is also important to note that FcRn inhibition is also an emerging therapeutic option in pemphigoid disease as it appears to have a favorable effect through the faster degradation of circulating antibodies. The inhibition with efgartigimod has been demonstrated to have a protective effect on the development of severe skin symptoms in both EBA and BP mouse models as well as on dermo-epidermal separation in a human 3D skin equivalent model (61–63). A clinical trial of efgartigimod was also initiated (BALLAD+ study [NCT05681481]), which reached phase 3 and showed effectiveness but unfortunately was subsequently discontinued as it failed to show statistically significant superior effectiveness over corticosteroid treatment alone.

### 3.2 Downstream signaling following Fcγ-receptor activation

#### 3.2.1 The role of Src-family tyrosine kinases

Various non-receptor tyrosine kinases play a key role in the signal transduction processes following immune complex recognition by Fcγ-receptors. They include Src-family tyrosine kinases, which are activated upon the ligand binding of activating Fcγ-receptors or integrins (64, 65). The Src tyrosine kinase family comprises a total of nine different proteins that are expressed in numerous different cell types. In myeloid cells, three of these, Hck, Fgr, and Lyn, have typically the highest expression (66). Following Fcγ-receptor or integrin ligand binding, these enzymes can phosphorylate two tyrosine molecules on the ITAM motif of the receptor itself or on the FcR γ-chain associated with the receptor, which enables further downstream signal transduction. The roles of these three Src-family kinases characteristic of myeloid cells have a significant overlap (65). In the absence of one of the three kinases, the other two are usually able to take over its role, and so the cell functions associated with Fcγ receptor activation, such as phagocytosis, ROS production, degranulation, and cytokine production, are only slightly affected. In contrast to the single knockouts, *Hck*^−/−^*Fgr*^−/−^*Lyn*^−/−^ triple knockout cells show complete defects in these cell functions (8, 65).

Our research group investigated the role of the three myeloid Src-family kinases (Hck, Fgr and Lyn) in the passive antibody transfer mouse model of EBA and found that triple knockout mice were completely protected from the development of severe skin symptoms, while the inflammatory cytokine levels and the infiltration of inflammatory cells were also abrogated in the ears of the animals (8). Furthermore, neutrophils of these mice also failed to conduct several effector responses induced by IgG immune complexes, such as ROS production, spreading, and cytokine production, while the *in vivo* migration ability of the cells remained intact (8, 9).

#### 3.2.2 Spleen tyrosine kinase

Spleen tyrosine kinase (Syk) is another non-receptor tyrosine kinase enzyme, which was first examined in B cells and was shown to play an important role in B-cell development and B-cell receptor signaling (67). Subsequently, it was also demonstrated that Syk has a high expression in myeloid cells and plays a key role in FcγR and integrin signaling (68, 69). Due to its SH2 domains, it can bind to the FcR γ-chain ITAM motifs phosphorylated by Src-family tyrosine kinases and then induce various cellular responses by phosphorylating downstream molecules (70). The important role is also demonstrated by the fact that in the absence of Syk, neutrophils fail to perform a number of effector cell responses upon FcγR or integrin stimuli, such as cytokine production, ROS production, and degranulation, while these cells preserve their migratory capacity (68, 69, 71). Although there are currently no clinically available drugs with high specificity for inhibiting Syk tyrosine kinase, the small molecule inhibitor fostamatinib which has an inhibitory effect on Syk is currently used in certain cases of chronic immune thrombocytopenia (72).

The importance of Syk tyrosine kinase was investigated in pemphigoid disease models, as well. Our research group and others examined the effect of Syk deficiency on disease development using the passive model of EBA (9, 10). These experiments revealed that, similarly to *Hck*^−/−^*Fgr*^−/−^*Lyn*^−/−^ triple knockout animals, mice lacking Syk in their hematopoietic compartment were also completely protected against the development of severe skin lesions, and their skin showed neither leukocyte infiltration nor the inflammatory cytokine environment characteristic of the disease (9). Moreover, the neutrophils of these Syk deficient mice were also completely defective in several effector functions (ROS production, spreading, cytokine production) (9), which prompted us to examine the effect of neutrophil-specific Syk deficiency in the EBA model using the Cre-lox system. The results showed that Syk deficiency induced only in the neutrophil compartment is also sufficient to achieve complete protection of these animals (11). To assess the role of Syk in human samples, we then performed experiment in the ex vivo skin separation assay, demonstrating that granulocyte-mediated skin separation induced by rabbit IgG-s against collagen VII protein can also be fully inhibited using the Syk-specific inhibitors entospletinib and lanraplenib (11). The Syk inhibitor entospletinib was also effective in a fully human ex vivo skin separation assay of bullous pemphigoid (12), demonstrating that Syk inhibition might be an attractive strategy in pemphigoid diseases.

#### 3.2.3 Phospholipase Cγ2

Phospholipase Cγ2 is an intracellular enzyme belonging to the large family of phospholipases. In this protein family there are two isoforms of PLCγ enzymes, PLCγ1 and PLCγ2. The former is known to be important in T cell signaling, while the second isoform was first identified in various processes of B cells (73). It has been shown that PLCγ2 is also dominant in myeloid cells and, like Src and Syk tyrosine kinases, plays an important role in the signaling of Fcγ and integrin receptors, where its activation is located downstream of Syk activation and is crucial for several neutrophil effector responses upon immune complex activation (73, 74).

Investigating the role in the EBA passive mouse model, the absence of PLCγ2, similar to that observed by *Hck*^−/−^*Fgr*^−/−^*Lyn*^−/−^ triple knockouts and Syk knockouts, results in complete protection in mice, reducing the infiltration of leukocytes and the release of inflammatory cytokines in the skin of the animals (75). Furthermore, by the neutrophils of PLCγ2-deficient mice, several effector responses induced by collagen VII immune complexes are impaired. Although there is currently no known specific inhibitor of PLCγ2, our group investigated the effect of a general PLC inhibitor (U73122) on ex vivo skin separation induced by mouse collagen VII IgG (75). The inhibitor completely abrogated the development of granulocyte-mediated dermo-epidermal separation in this model.

#### 3.2.4 CARD9

Caspase recruitment domain-containing protein 9 (CARD9) is an intracellular adapter protein that is highly expressed in myeloid cells (76). Our research group has shown that CARD9-deficient neutrophils differ significantly from WT cells in their gene expression, and in CARD9 deficient neutrophils, following Fcγ receptor activation, the production of various chemokines and cytokines (MIP1-α, MIP-2, IL-1β) are diminished upon immune complex stimuli, while the cells preserve their short-term responses independent of gene expression changes such as the production of ROS and leukotriene B_4_ (77).

Our group also examined the effect of CARD9 deficiency *in vivo* in the EBA passive inflammatory model, where CARD9 knock out mice showed moderate symptoms compared to wild type animals after antibody administration, suggesting that the production of inflammatory cytokines plays a significant role in pathogenesis (77). Nevertheless, the inhibition of the establishment of the inflammatory microenvironment in the skin alone was not sufficient for a complete protection.

**Figure 3:**
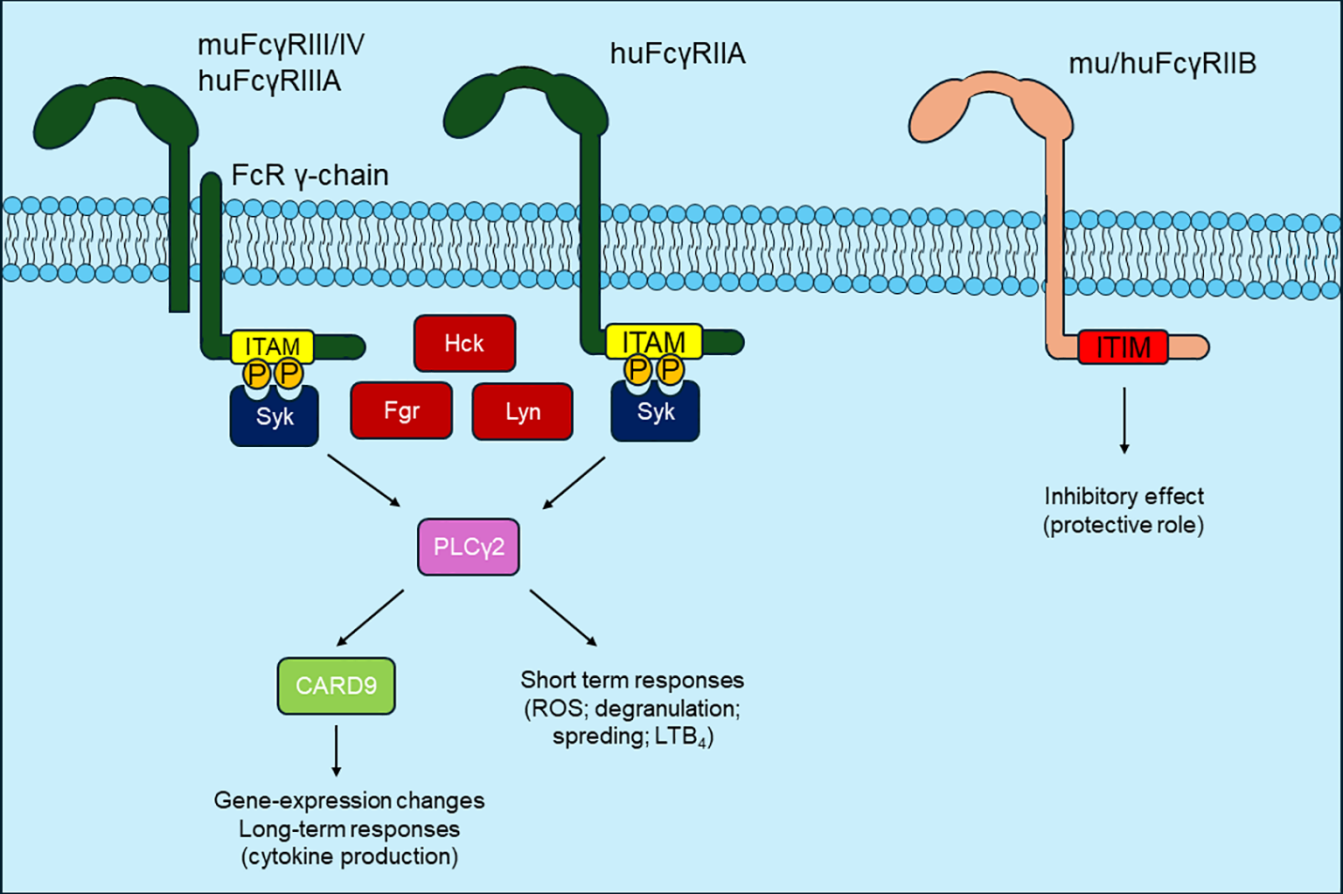
Fcγ-receptor signaling in pemphigoid diseases. Fcγ-receptors play an essential role in the recognition of IgG immune complexes. These receptors include activating receptors, which either have their own ITAM motif or are directly linked to an ITAM-containing γ-chain. In the signalling of these receptors certain Src-type tyrosine kinases, Syk tyrosine kinase and Phospholipase Cγ2 have an important role. The activation of these proteins directly leads to short-term effector responses (ROS production, degranulation, LTB_4_ production) and to longer-term gene expression changes in a CARD9-dependent manner, strongly contributing to dermo-epidermal separation. Another group of FcγRs are inhibitory receptors containing the ITIM motif, which might have a protective role in pemphigoid diseases.

Overall, as a summary of these experiments, it appears that, while inhibition of the FcRγ/Src-family/Syk/PLCγ2 signaling pathway induces complete protection against disease development, presumably (seeing the protective effect upon neutrophil-specific Syk deletion alone) by inhibiting several effector functions of neutrophils, the inhibition of CARD9 downstream from the above pathway only results in partial protection. The reason can be, that CARD9 activation contributes to the disease primarily by regulating the gene expression of neutrophils, thereby mediating the production of various cytokines and the development of the inflammatory microenvironment characteristic of the disease, but has no effect on short-term neutrophil responses (**Figure 3**). Based on this duality (inhibition of rapid inflammatory cell responses in the absence of FcRγ/Src-family/Syk/PLCγ2 and inhibition of slower gene expression changes in the absence of CARD9), a combined treatment strategy (e.g., simultaneous inhibition of Syk and CARD9) may be worth considering in the future.

## 4 JAK inhibitors in the treatment of pemphigoid diseases

### 4.1 General characteristics of JAK tyrosine kinases

Similar to Src-family tyrosine kinases and Syk, JAK-family tyrosine kinases also belong to the large superfamily of non-receptor tyrosine kinases. In total, there are four proteins in this family: JAK1, JAK2, JAK3, and TYK2, which are involved in signal transduction of receptors for more than 50 different cytokines, such as interleukins, interferons and colony-stimulating factors. While JAK1, JAK2, and TYK2 are very broadly expressed in many different cell types, the expression of JAK3 is limited to cells of hematopoietic origin. Typically, these enzymes act as dimers in the cytoplasm (in most cases heterodimers, less commonly homodimers, which is characteristic of JAK2) (78, 79).

The signal transduction associated with JAK tyrosine kinases is evolutionary conserved. Following ligand binding to the appropriate receptors, JAK proteins undergo a conformational change, resulting in JAK activation and the trans-autophosphorylation of tyrosine residues by the kinases itself. As a result, the kinase activity of the molecules increases even further, and they also phosphorylate the corresponding tyrosine molecules of the receptors. Following that, the phosphorylated receptors will be capable of recruiting signal transducer and activator of transcription (STAT) proteins (a total of 7 different STATs have been described: STAT1, STAT2, STAT3, STAT4, STAT5a, STAT5b, and STAT6), which then enter the cell nucleus and induce gene-expression changes and epigenetic alterations (79).

Malfunction of the JAK/STAT signaling pathways due to genetic mutations can lead to various diseases including malignancies (e.g., JAK2 gain of function mutation in polycythemia rubra vera (80)) and immunodeficiencies (e.g., JAK3 loss of function in severe combined immunodeficiency (81)). On the contrary, targeting JAKs with small-molecule inhibitors has been identified as an effective therapeutic solution in several autoimmune diseases such as rheumatoid arthritis and systemic lupus erythematosus (79). Several of these inhibitors already used in the clinical practice are now available and show different specificities towards the various JAK-family members (**Table 3**). In the case of pemphigoid diseases, there is an increasing number of studies demonstrating beneficial effects of various JAK inhibitors in patients when conventional therapies are either contraindicated or ineffective.

**Table 3:** JAK inhibitors in the clinical practice.

Drug	Primary target(s)	IC50 in cell free assay (nM)					Indication	Case studies
		JAK1	JAK2	JAK3	TYK2	Others		
Tofacitinib	JAK1, JAK2, JAK3	112	20	1	34	–	Rheumatoid arthritis, Psoriatic arthritis, Ankylosing spondilytis, Ulcerative colitis, Polyarticular course juvenile idiopathic arthritis (FDA, EMA)	BP (91, 94, 96, 99, 136), EBA (87), MMP (105, 109), LIABD (111), LPP (114, 116), anti-p200 pemphigoid (110)
Baricitinib	JAK1, JAK2	5.9	5.7	560	53	–	Rheumatoid arthritis, COVID19 (FDA), Atopic dermatitis, Alopecia areata, Juvenil idiopathic arthritis (EMA)	BP (97, 98, 100, 101), MMP (100, 104, 106, 109), LPP (113)
Upadacitinib	JAK1	47	120	2,300	4,700	–	Rheumatoid arthritis, Psoriatic arthritis, Ankylosing spondilitis, spondyloarthritis, giant cell arteritis, ulcerative colitis, Chron’s disease (FDA, EMA)	BP (90, 92, 137), EBA (88), MMP (108), LIABP (112), LPP (115)
Abrocitinib	JAK1	29	803	>10,000	1,253	–	Atopic dermatitis (FDA, EMA)	BP (93, 95, 103), MMP (107), LPP (115)
Filgotinib	JAK1, JAK2	10	28	810	116	–	Rheumatoid arthritis, ulcerative colitis (EMA)	n.a
Ruxolitinib	JAK1, JAK2	3.3	2.8	428	19	–	Primary and secondary myelofibrosis, pediatric atopic dermatitis (FDA), Polycythemia rubra vera, Graft versus host disease (EMA)	n.a
Fedratinib	JAK2 (FLT3)	105	3	1,002	n.a.	15 (FLT3)	Primary and secondary myelofibrosis (FDA, EMA)	n.a
Pacritinib	JAK2 (FLT3, IRAK1)	1280	19	520	50	22 (FLT3); n.a. (IRAK1)	Primary and secondary myelofibrosis (FDA)	n.a
Momelotinib	JAK1, JAK2	11	18	155	n.a.	–	Primary and secondary myelofibrosis (FDA, EMA)	n.a
Ritlecitinib	JAK3 (TEC)	33	>10,000	>10,000	>10,000	n.a. (TEC)	Alopecia areata (FDA, EMA)	n.a
Deucravacitinib	TYK2	1	n.a.	n.a.	0.2	–	Psoriasis (FDA, EMA)	n.a
Delgocitinib	JAK1, JAK2	2.8	2.6	13	58	–	Atopic dermatitis (FDA, EMA)	n.a

Obtained from MedChem Express and Selleckchem. n.a,not available.

### 4.2 JAK inhibitors in the treatment of pemphigoid diseases

Despite the numerous existing animal models of pemphigoid diseases (82), there is currently a lack of experimental results with JAK inhibitors from such models in the literature, presumably at least partly because of the differences in the cytokine profiles in mouse models and human diseases. Especially, in dominantly neutrophil-derived mouse models, key eosinophil-derived cytokines characteristic of human BP are only present in low quantity (as IL-5 is not overexpressed in the EBA model (10). To bridge this gap, transgenic mice expressing these mediators could be useful in the investigation of the role of JAK kinases in the future. A clear limitation of such experiments is that there are substantial differences between human autoimmune subepidermal blistering skin diseases and their mouse models in terms of active and passive immunization, ease of induction, as well as clinical and histological signs (e. g. presence and involvement of eosinophils). A potential strategy to emphasize the role of eosinophils would be the use of an IL-5 transgenic mouse strain. Despite the current lack of animal experiments, the potential beneficial effect of targeting JAK tyrosine kinases is, however, suggested by several human data. Investigating the skin lesions of patients with BP, it was demonstrated that the expression of enzymes of the JAK/STAT signaling pathways are elevated compared to control samples (83). Furthermore, the potential efficacy of JAK inhibitors is supported by the fact that the two targets of dupilumab (the only approved targeted therapy available in BP) are IL-4 and IL-13, the receptors of both of which involve JAK enzymes in their signaling (79). In the case of IL-4, JAK1 and JAK3 are crucial players in receptor signaling, while in the case of IL-13 (whose prominent role has been recently demonstrated by transcriptional studies (84)), the involvement of TYK2 is essential. In addition, several other cytokines that signal through JAKs, such as IL-8 and IL-6 have been described as highly represented in pemphigoid lesions, and might play an important role in the pathogenesis (85, 86).

Although there are no controlled clinical trials conducted on JAK inhibitors in pemphigoid diseases, case studies have reported favorable effects in several cases with tofacitinib (JAK1/3 inhibitor), baricitinib (JAK1/2 inhibitor), upadacitinib (JAK1/3 inhibitor), or abrocitinib (JAK1/3 inhibitor). In individual patients with EBA, there are two reported cases of JAK inhibitor treatment (one patient treated with tofacitinib (87) and another with upadacitinib as a bridging therapy to rituximab (88)), while in BP, since 2022 a total of 26 patients have been reported in case studies to be treated with various JAK inhibitors (mostly tofacitinib, and in a couple of cases baricitinib, upadacitinib, or abrocitinib) (87, 89–102) and there is a larger retrospective cohort study reporting on 21 patients treated with abrocitinib in combination with low-dose corticosteroid (103). In one case study with tofacitinib treatment, a severe hemilateral retinal occlusion was reported by a patient with previously treated glaucoma, ultimately leading to the withdrawal of the JAK inhibitor underlining the importance of careful consideration, especially in case of older patients, of potential risk factors listed in the “black box warning” of these drugs (thromboembolism, infection, malignancies). Nevertheless, in most individual cases, the therapy turned out to be highly effective and safe, resulting in no significant side effects, while in the cohort study, complete remission was achieved in 52% of the cases with abrocitinib combined with low dose corticosteroid treatment. In addition, a total of 7 studies have been conducted on patients suffering from mucous membrane pemphigoid, another rare and especially difficult-to-treat variant of the pemphigoid group, in which patients were treated with tofacitinib, baricitinib, or, in one case each, abrocitinib and upadacitinib (104–109). Some case studies were also conducted with different JAK inhibitors in even rarer pemphigoid diseases, including anti-p200 pemphigoid (110), linear IgA bullous dermatosis (111, 112) and lichen planus pemphigoid (113–116). In most cases, these studies report favorable effects, in a number of cases even complete remission, with an acceptable side effect profile in patients for whom conventional therapeutic options were not available. Further information on the role of JAKs and the efficacy of JAK inhibitor treatment in autoimmune bullous diseases are available from two excellent recent reviews (13, 117).

Overall, JAK inhibitors are potential candidates to serve as excellent therapeutics in the treatment of pemphigoid diseases, but randomized controlled clinical trials should be conducted in the future to assess superior effectiveness and safety compared to conventional therapies.

## 5 Other non-receptor tyrosine kinases

While the relevance of FcγR activation-related non-receptor tyrosine kinases (Src-family kinases and Syk) and JAK family kinases has been shown by numerous mouse and human experimental and clinical results, very little information is currently available on the role of other non-receptor tyrosine kinase families (Abl, Tec, Fak, and other smaller families: Csk, Fes, Ack) in pemphigoid disease pathogenesis.

The role of the Tec family kinase Bruton’s tyrosine kinase (Btk) has been studied extensively in the pemphigus group of autoimmune blistering skin diseases (118). Here, the efficacy of Btk inhibition has been demonstrated both in animal experiments (119, 120) and in clinical case studies (121, 122). These results were so promising that a clinical trial with rilzabrutinib was initiated (123) (Belive study [NCT02704429] and Pegasus study [NCT03762265]), and a controlled clinical trial is currently underway to evaluate the efficacy of tirabrutinib, another small-molecule Btk inhibitor, in steroid-resistant pemphigus (Brilliant study [NCT06696716]). Although no experimental results are available with Btk inhibitors in either BP or EBA, their efficacy in pemphigus raises the possibility that these drugs might also have therapeutic benefit in pemphigoid diseases.

Also, little information is available on the role of tyrosine kinases belonging to the Abl family. The only data available on the treatment of bullous pemphigoid is that obtained with imatinib, a small-molecule inhibitor developed for the Bcr-Abl fusion protein. In the literature a case report can be found about a patient with hypereosinophilia associated with bullous pemphigoid, who responded well to imatinib treatment (124). However, another patient with chronic myeloid leukaemia developed a BP-like disease after imatinib therapy (125), showing a controversy over the potential efficacy of the drug in pemphigoid.

Similar to Btk, data about the role of the focal adhesion kinase (Fak) is only available for the pemphigus disease group, as Fak is known to be highly expressed in keratinocytes associated with pemphigus vulgaris and foliaceus (126). In addition, its inhibition has been shown to be effective in preventing the development of blistering skin lesions in an animal model of pemphigus vulgaris (127). However, there are no data from human studies yet, nor are there any data on BP or EBA disease models, but its apparent role in experimental pemphigus suggests that investigating the role of Fak in pemphigoid diseases may also reveal important novel information.

As far as we know, there is no public information on the role of smaller non-receptor tyrosine kinase families (Csk, Fes, Ack) in pemphigoid diseases.

Overall, while the clinical applicability of the Src, Syk, and JAK tyrosine kinases discussed above is expected to be investigated in the coming years, basic research is needed to investigate the potential role of other non-receptor tyrosine kinases in pemphigoid disease. Hopefully the upcoming years the experimental results will initiate several new controlled clinical trials (**Table 4**) providing better therapeutic options for the patients.

**Table 4:** Current and completed clinical trials of kinase inhibitors and related compounds in pemphigoid diseases.

Trial identifier	Disease	Drug	Trial phase	Status	Results
NCT05267600 and NCT05681481	BP	efgartigimod	Phase 2/3 and phase 3	Completed/Terminated	Superior effectiveness has not been proven
NCT06561256	BP	Various JAK inhibitors	Observational study	Completed	No publication is available
No NCT number is available	BP	abrocitinib	Cohort study	Completed, publication is available (103)	Superior effectiveness of abrocitinib combined with low dose corticosteroid (11 patients [52.4%] complete remission) versus azathioprine combined with low dose corticosteroid (2 patients [9.1%] complete remission) has been found (p=0.003)
NCT05263505	MMP	baricitinib	Phase 2	Terminated prematurely	Challenges with patient recruitment
NCT06834035	EBA	efgartigimod	Phase 1/2	Not yet recruiting	-

## 6 Conclusion

The successful use of tyrosine kinase inhibitors in numerous hematological malignancies and the rise of JAK inhibitors in many types of autoimmune diseases have aroused interest in the role of tyrosine kinases in pathological processes in the field of immunology. This review examined the current understanding of the role of tyrosine kinase signaling pathways important in the development of pemphigoid diseases within the group of autoimmune blistering skin diseases. In recent years, we and others have reported numerous findings regarding the signal transduction initiated by the recognition of immune complexes via activating Fcγ-receptors, in which pathway Src-family tyrosine kinases and Syk are attractive targets, as their role seems to be very important in both animal and human experimental systems. Similarly, JAK tyrosine kinases are also attractive therapeutic targets, as numerous cases, successfully treated with various JAK inhibitors were published in recent years. In addition to the recent discoveries in basic research, dermatological diseases offer good opportunities for therapeutic developments, as topical application enables a favorable side effect profile, avoiding systemic drug effects. However, the important role of the skin and mucous membranes, as vital barriers to the outside world, also pose unique demands and challenges for the local application of new therapeutics, several small-molecule tyrosine kinase inhibitors show great potential for adequate skin penetration having small molecular weight and high lipophilicity (as Syk inhibitors entospletinib and the active metabolite of fostamatinib [R406], the multi-kinase inhibitor dasatinib, or several JAK inhibitors, some already exist in topical formulation [deucravacitinib]). In addition, combination therapies targeting several tyrosine kinase signaling pathways may also be a suitable future therapeutic strategy. Finally, the role and potential targeting of the complement system is also a highly relevant issue in the therapy of autoimmune subepidermal blistering skin diseases. Overall, the experimental results of recent years and the continuous research into the better understanding of the pathomechanism of pemphigoid diseases will hopefully contribute greatly to the development of this particularly exciting field of drug development, leading to better treatment of these difficult-to-treat diseases.
